# Identifying sources of non-stationary neural ensemble dynamics

**DOI:** 10.1186/1471-2202-14-S1-P15

**Published:** 2013-07-08

**Authors:** Emili Balaguer-Ballester, Hamid Bouchachia, Christopher C Lapish

**Affiliations:** 1 School of Engineering and Computing, Bournemouth University, Bournemouth, BH12 5BB, UK; 2 Bernstein Center for Computational Neuroscience, ZI Mannheim-University of Heidelberg, Mannheim, J5, Germany; 3 Department of Psychology, Indiana University-Purdue University, Indianapolis, Indiana, 46202, USA

## 

In the traditional view on brain activity dynamics, the cognitive flow of information wanders through multiple stable states driven by task-dependent inputs [1-3]. This focus has been recently challenged both empirically and from the modeling perspective. For instance, experimental studies suggest that olfactory [4] and gustatory representations [5] can be understood as a sequence of temporally stable, attractor-like states; but such transient states are essentially transient and driven by stochastic fluctuations. Likewise, in several contemporary models, intrinsic activity fluctuations can drive default transitions between states [6,7].

It has been recently proposed that such transient states are basically shaped by anatomical connectivity and transitions between them occur even in the absence of external stimuli [8]: Noise enriches the dynamical repertoire of deterministic states; creating flexible 'ghost' attractors which enable the effective processing of task-related cognitive entities [7].

A different metaphor of transient brain dynamics was proposed by Rabinovich and colleagues [9]. In such model, transitions between states mapping cognitive entities is purely deterministic: The dynamical portrait of the model consists of successions of temporally stable states i.e. metastable saddle points linked by heteroclinic channels. Such dynamical objects are particularly reliable, but neural activity eventually switches between them even without the intervention of noise or external inputs.

In this work we develop an empirical criterion to discern whether observable neural ensemble activity can be originated by non-autonomous yet deterministic dynamical systems or rather by stochastic fluctuations between temporally attracting states. Towards this goal, we used in vivo multiple single-unit recording in rodent frontal cortex during a decision making task. Effective dynamics of neural activity is first empirically reconstructed in nonlinear state spaces [10]. Then, trajectory analyses enable us to differentiate systems driven by non-automatous dynamics from those driven by stochastic transitions.

## Conclusions

Underlying dynamics of recorded ensemble activity is probably driven by a slowly drifting, non-autonomous dynamical system containing low-order nonlinear interactions.
